# MicroProcSim: A Software for Simulation of Microstructure Evolution

**DOI:** 10.1007/s40192-025-00405-6

**Published:** 2025-06-23

**Authors:** Md Maruf Billah, Muhammed Nur Talha Kilic, Md Mahmudul Hasan, Zekeriya Ender Eger, Yuwei Mao, Kewei Wang, Alok Choudhary, Ankit Agrawal, Veera Sundararaghavan, Pınar Acar

**Affiliations:** 1 Department of Mechanical Engineering, Virginia Tech, Blacksburg, VA 24061 USA; 2 Department of Electrical and Computer Engineering, Northwestern University, Evanston, IL 60208 USA; 3 Department of Aerospace Engineering, University of Michigan, Ann Arbor, MI 48109 USA

**Keywords:** Crystal plasticity modeling, Processing simulation, Strain rate, Microstructure, ODF

## Abstract

Understanding the large deformation behavior of materials under external forces is crucial for reliable engineering applications. The mechanical properties of materials depend on their underlying microstructures, which change over time during deformation. Experimental observation of these processes is time-consuming and influenced by various conditions. Therefore, we developed MicroProcSim, a physics-based simulation tool to replicate the deformation process of cubic microstructures. MicroProcSim can predict the evolution of texture, represented by the orientation distribution function (ODF), over time under various loads and strain rates. This software package can be run on both Windows and Linux operating systems. Unlike conventional crystal plasticity finite element software, MicroProcSim offers a distinct advantage by rapidly generating deformed textures, as it bypasses incorporating grain morphology. Furthermore, comparisons with existing experimental and computational studies on texture evolution have demonstrated that this software seamlessly replicates real-world material processing conditions through a simple modification of a single input matrix.

**Editor’s Video Summary:**

The online version of this article (10.1007/s40192-025-00405-6) contains an Editor’s Video Summary, which is available to authorized users.

## Editor’s Video Summary

Summary (MP4)

## Introduction

The crystallographic texture of microstructures plays a pivotal role in determining the micro-scale characteristics of materials [1–3]. Controlling polycrystalline microstructures is essential in materials design. This is due to orientation-dependent material properties, such as stiffness and yield strength, which can vary as microstructures evolve during deformation [4–6]. Effective management of microstructures ensures the reliability of the desired material’s performance. A significant amount of research, both experimental and theoretical, has been devoted to understanding and predicting the development of crystallographic textures in metallic microstructures. Textures that emerge due to specific monotonic deformations in cubic polycrystals have been studied extensively [7–9]. Particular focus has been given to textures arising from uniaxial compression or tension, plane strain compression, and simple shear [10, 11]. The significance of these deformations lies in their application in standard mechanical tests such as channel die compression, uniaxial compression, and torsion [12]. These tests are pivotal for assessing the mechanical behavior of materials under large strains. Furthermore, these deformation modes indicate the stresses encountered during various industrial forming processes, including extrusion, wire drawing, and rolling [13]. The ability to replicate these stress conditions in laboratory settings is crucial for predicting material performance, discovering new materials, and optimizing industrial manufacturing processes [14].

Various computational approaches have been developed to model how crystalline materials deform and how their textures evolve under different loading conditions. At the foundational level, there are simpler formulations, such as the original Taylor model [15] and its relaxed variants [16, 17]. These methods are straightforward and can provide valuable insights into the deformation behavior of crystalline aggregates under uniform strain assumptions. Intermediate complexity approaches include cluster-based frameworks such as Lamel model [18–20], advanced grain interaction (GIA) method [21–23], and relaxed grain cluster (RGC) schemes [24, 25]. These methods strike a balance between computational efficiency and capturing more realistic material behaviors, making them suitable for many practical applications. Moving toward more advanced methods, the self-consistent family of models integrates additional material behaviors. These include viscoplastic self-consistent (VPSC) [26, 27], elastic-rigid-plastic self-consistent (EPSC) [28, 29], and elastic-viscoplastic self-consistent (EVPSC) [30, 31] formulations. By considering interactions between individual grains and their surrounding matrix, these models offer improved accuracy in predicting texture evolution and anisotropic material responses. At the cutting edge of crystal plasticity modeling are full-field approaches, such as spectral methods leveraging fast Fourier transforms [32] and crystal plasticity finite element methods (CPFEM) [33]. These sophisticated techniques capture detailed microstructural responses, accommodating complex boundary conditions and local heterogeneities, albeit at a higher computational cost. Each method presents unique strengths, with their applicability determined by the specific phenomena of interest and the computational resources available. By selecting the appropriate approach, researchers can tailor their models to achieve the best compromise between accuracy and efficiency for their investigations.

In the field of texture development modeling, the orientation distribution function (ODF) has been established as an efficient and convenient method of representing microstructural texture because it serves as a one-point probability descriptor of grain orientation while simplifying complex analyses [34–41]. This technique has received attention because of its ability to provide a detailed quantitative description of textures, enhancing our understanding and application of ODFs [42–45]. The shift toward ODF-based techniques for microstructure modeling reflects their capability in capturing the complexity of texture evolution. In particular, we have utilized existing established finite element approaches in the development of MicroProcSim. This software utilizes the finite element-based ODF scheme with piecewise polynomial interpolation functions representing the texture over Rodrigues orientation space. This representation offers several key benefits. The simplicity and localized nature of these polynomial functions allow them to effectively model sharp textures. Furthermore, the finite element framework facilitates the construction of texture transformation analogs, such as interpolation, differencing, and projection for ODFs [12]. For example, the piecewise polynomial ODFs driven from deformation evolution are evaluated by employing well-established finite element methods to solve the ODF conservation equation for hyperbolic conservation laws. Furthermore, Rodrigues parameters uniquely map each orientation to a specific position within the Rodrigues fundamental region, ensuring a single, unambiguous representation [46].

The primary strength of CPFEM lies in its ability to explicitly capture the mechanical interactions among crystals within a polycrystal, without relying on homogenization assumptions. By incorporating constitutive formulations at the level of individual shear systems, CPFEM offers a framework capable of modeling physics-based, multiscale internal-variable plasticity, including various size-dependent effects and interface mechanisms [47, 48]. It also enables detailed access to both intra- and inter-grain deformation behaviors, making it particularly valuable for investigating grain boundary phenomena [49, 50]. In contrast, ODF-based methods offer a more efficient and simplified alternative, particularly suited for large-scale applications or cases where a statistical representation of texture suffices. This formulation cannot account for deformation mechanisms e.g., grain boundary sliding, non-crystallographic rotation, and twinning, which significantly influence reorientation in many crystal plasticity problems, limiting its applicability in capturing complex microstructural evolution accurately. The current ODF-based methods are particularly advantageous when global texture evolution rather than local stress–strain distributions is the primary focus. The statistical nature of ODFs also makes them well-suited for uncertainty quantification and inverse design problems where computational efficiency is paramount [51, 52].

In order to advance the deformation simulation of the materials, we have developed a software package called, ‘MicroProcSim,’ which can capture the texture evolution of the cubic microstructures under different loading conditions. This software is capable of simulating a wide range of material processing conditions, where various deformation modes contribute to altering the microstructural texture. Additionally, the strain rate and initial microstructural texture can be customized to replicate real experimental conditions. However, this article shows only three pure normal strain cases, three pure shear strain cases, and three plane strain deformation cases. The development of MicroProcSim revolutionizes materials science and engineering by enabling efficient simulation of microstructural texture evolution under various loading conditions. It overcomes experimental limitations, accelerates research, and reduces resource demands, allowing researchers to address advanced questions like optimizing material properties and process parameters. Notably, several studies [3, 10, 11, 53–55] have already published work utilizing MicroProcSim, demonstrating its widespread adoption and impact.

The article is structured as follows: the underlying physics behind the code development for the presented software will be discussed in section "Software Background", along with an illustrative guideline for operating the software. Section "Software Architecture" describes the architecture of this software. Following this, a few examples of microstructure evolution under different process conditions will be presented in section "Illustrative Examples". Then section "Extension of the Code to Different Operating Systems" delineates the extension of the software that runs on different operating systems. Later, section "Comparative Analysis" will present several experimental and computational studies that report texture evolution. These studies will be compared with the textures generated by MicroProcSim under similar deformation processes. Then, a summary of the computational costs associated with the example simulations will be provided in section 'Computational Costs". Finally, comprehensive conclusions will be drawn in section "Conclusion".

## Software Background

### Representation of Crystallographic Orientations

This software uses ODFs as a probability descriptor to represent the crystallographic textures of metallic microstructures. The ODFs essentially relate to the volume densities of the crystallographic orientations and are utilized to calculate the homogenized mechanical properties through local finite element discretization methods. In this work, the axis-angle parametrization of Rodrigues orientation space is employed, where the axis of rotation is scaled as ${\textbf{r}} = {\textbf{n}} \tan (\theta /2)$; here, ${\textbf{n}}$ and $\theta$ represent the rotation axis and the angle of rotation, respectively [12]. The lattice orientation, ${\textbf{R}}$, and the Rodrigues parameter, ${\textbf{r}}$, can be expressed by the relationship in Eq. (1) [9].

1 $$\begin{aligned} {\textbf{R}}=\frac{1}{1+{\textbf{r}}.{\textbf{r}}}({\textbf{I}}(1-{\textbf{r}}.{\textbf{r}})+2({\textbf{r}}\otimes {\textbf{r}}+{\textbf{I}}\times {\textbf{r}})) \end{aligned}$$

**Fig. 1:**
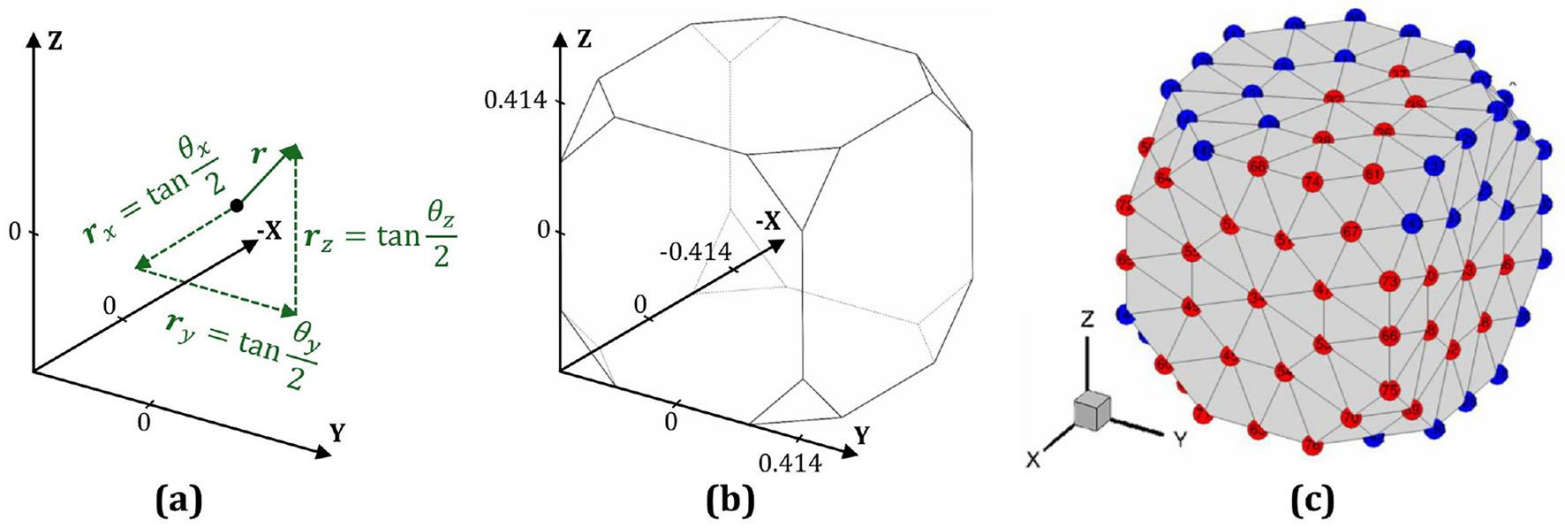
**a** Schematic representation of the Rodrigues parameter (${\textbf{r}}$) defined in terms of orientation angles, **b** fundamental Rodrigues orientation space for an FCC crystal, and **c** finite element mesh representation of the Rodrigues orientation space, with ODFs specified at nodal points. Red points denote independent ODFs, while blue points indicate dependent ODFs

The vector (${\textbf{r}}$) can be represented as shown in Fig. 1a which operates in a three-dimensional space, offering advantages over two-dimensional stereographic projections. The geometry of the Rodrigues projective representation is influenced by both the symmetry of the object or lattice being studied and the specific characteristics of the Rodrigues space. Various researchers extensively documented the mathematical principles underlying Rodrigues space which can be accessed through the referred articles [56–58]. In this representation, orientation vectors are constrained by a maximum magnitude, which corresponds to the highest possible orientation achievable in a particular direction for a given symmetrical volume. When mapped in three dimensions, these vector endpoints form intricate polyhedral shapes as illustrated in Fig. 1b, contrasting with the spherical forms used in stereographic projections or the rectangular configurations seen in Euler angle coordinate systems [59]. The dimensions of the cubic Rodrigues fundamental space are determined by its constituent vectors. Along the axes, the shortest vectors that reach the surface have a magnitude of $\tan (45^\circ / 2) = \sqrt{2} - 1$. The vectors extending to the truncating triangles measure $\tan (60^\circ / 2) \approx 0.58$ in magnitude. For cubic symmetry, the maximum rotation angle occurs around a $(1,1,\sqrt{2} - 1)$ axis, with its vectors having a magnitude of $\tan (62.8^\circ / 2) \approx 0.61$. Within the fundamental zone, each point corresponds to a unique orientation relative to a reference frame lacking symmetry, collectively forming what is known as the reduced orientation set.

The orientation spaces of polycrystalline face-centered cubic (FCC) structures are simplified due to reduced orientation set or crystal symmetries; among the 145 elements of the ODF, 76 remain independent representing 76 unique orientations, as shown in Fig. 1c [38, 60]. By using a local finite element method, the fundamental region is discretized, which involves *N* independent nodes and $N_{\text {elm}}$ finite elements, each with $N_{\text {int}}$ integration points. The unique ODF value at each nodal point in the mesh is intricately linked to the volume density of the corresponding crystallographic orientation. Subsequently, the set of ODFs (*A*) for any microstructure over the fundamental region must be normalized to unity, as expressed in Eq. (2) where $w_m$ is the integration weight associated with the $m$-th integration point, and $|J_n|$ is the Jacobian determinant of the $n$-th element [52, 61, 62].

2 $$\begin{aligned} \int _\Omega A({\textbf{r}},t)d\nu =\sum _{n=1}^{N_{elm}}\sum _{m=1}^{N_{int}}A(\mathbf {r_m},t)w_m|J_n|\frac{1}{\left( 1+\mathbf {r_m}.\mathbf {r_m}\right) ^2}=1 \end{aligned}$$

### Microstructural Texture Evolution

The applied force can change the microstructural orientations, represented by the ODFs, during deformation. This evolution occurs within the constraints of volume normalization and the conservation of ODFs from the initial time ($t = 0$) to the final time ($t = t$) [10]. The conservation rule for crystallographic orientations can be described by the following Eq. (3), using the Eulerian rate form. In this equation, the reorientation velocity ($\upsilon$) is crucial for the evolution of ODFs and can be expressed by Eq. (4) [53]. Here, the spin vector is denoted by $\omega$, which is a vector form of the tensor ${\dot{{\varvec{R}}}}^e{{\varvec{R}}}^{e^T}$. According to the crystal plasticity model, elastic deformation gradient ${{\varvec{F}}}^e$ assists to find ${{\varvec{R}}}^e$ through ${{\varvec{F}}}^e={{\varvec{R}}}^e{{\varvec{U}}}^e$, where ${{\varvec{U}}}^e$ represents the polar decomposition’s unitary tensor [63].

3 $$\begin{aligned} & \frac{\partial A({\textbf{r}},t)}{\partial t}+\nabla A({\textbf{r}},t)\cdot \upsilon ({\textbf{r}},t)+A({\textbf{r}},t)\nabla \cdot \upsilon ({\textbf{r}},t)=0 \end{aligned}$$

4 $$\begin{aligned} & \upsilon ({\textbf{r}},t)=\frac{1}{2}(\omega +(\omega \cdot r)r+\omega \times r) \end{aligned}$$

Furthermore, the reorientation velocity ($\upsilon$) is utilized to form the macro velocity gradient (${\textbf{L}}$) as formulated in Eq. (5). The microstructure constitutive model is used as the governing equation of ODF evolution, which can be simply expressed in terms of the macro velocity gradient. On the other hand, Taylor’s macro-micro hypothesis [15] concludes that the reorientation velocity can be linked to the velocity gradient, where the macro velocity gradient should be equal to the crystal velocity gradient. Subsequently, the reorientation velocity is calculated from a constitutive model which is rate-independent. This means that the final texture $A({\textbf{r}},t)$ is derived from the initial texture $A({\textbf{r}},0)$ by employing the previously mentioned finite element discretization method in Rodrigues space, along with this constitutive model.

5 $$\begin{aligned} & {\textbf{L}}={\textbf{S}}+{\textbf{R}}\sum _\alpha {\dot{\gamma }}^\alpha {\bar{\textbf{T}}}^\alpha {\textbf{R}}^T \end{aligned}$$

6 $$\begin{aligned} & {\dot{\gamma }}^\alpha =\quad {\dot{\gamma }}^0\left( \frac{\tau ^\alpha }{s}\right) ^{\frac{1}{m}}\operatorname {sgn}\left( (\frac{\tau ^\alpha }{s})\right) \end{aligned}$$

Every process condition results in a distinct deformation category, such as tension, compression, or shear, which can be defined with the timeframe of simulation in the input of the software. Ultimately, the macro velocity gradient controls the overall deformation process of crystal plasticity, which is used to evaluate the evolution of the ODF. However, to achieve a specific final texture from a given initial texture, the velocity gradient can be treated as an unknown variable. The relationship between lattice rotation (${\textbf{R}}$), lattice spin (${\textbf{S}}$), and macro velocity gradient (${\textbf{L}}$) can be formulated as shown in Eq. (5). The Schmid tensor and rate of shear of the $\alpha ^{th}$ slip system are represented by ${\bar{\textbf{T}}}^\alpha$ and ${\dot{\gamma }}^\alpha$, respectively. The shearing rate is defined by the Eq. (6), where *s* is the slip system hardness (considered uniform across all slip systems), *m* is the strain rate sensitivity, ${\dot{\gamma }}^0$ is a reference shearing rate, and $\tau ^\alpha$ is the resolved shearing rate on slip system $\alpha$ [9]. The material parameters ${\dot{\gamma }}^0$, *m*, and *s* were considered as $1 \textrm{s}^{-1}$, $0.05$, and $27.17 \textrm{MPa}$, respectively. The macro velocity gradient expression essentially comprises two primary components: one is the lattice spin, regarding the deformation gradient (${\varvec{F}}$), which can be further decomposed into elastic (${\varvec{F}}^e$) and plastic (${\varvec{F}}^p$) deformation where ${\varvec{F}} = {\varvec{F}}^e {\varvec{F}}^p$. The other term represents the rotated plastic velocity gradient resulting from the summation of shearing of all slip systems, expressed by ${\textbf{R}}\sum _\alpha {\dot{\gamma }}^\alpha {\bar{\textbf{T}}}^\alpha {\textbf{R}}^T$. However, the macro velocity gradient can be expressed in a simple matrix form as shown in Eq. (7), where the decomposition of the velocity gradient tensor reveals several fundamental physical processes. When broken down mathematically, distinct terms emerge that each correspond to different types of motion. The initial component describes tensile deformation along the *x* axis, followed by a term capturing rolling process along the *y* axis. The remaining three components represent pure shear deformation occurring in different spatial orientations. By combining these basic elements in varying proportions (adjustment of the process parameters $\alpha _{1}$, $\alpha _{2}$, $\alpha _{3}$, $\alpha _{4}$, and $\alpha _{5}$), any incompressible deformation pattern and deformation strain rate can be mathematically represented. This framework provides a complete basis for describing how the material can distort while maintaining a constant volume. The detailed derivation can be accessed from the referred article [9].

7 $$\begin{aligned} & {\textbf{L}}=\alpha _1\begin{bmatrix}1& 0& 0\\ 0& -0.5& 0\\ 0& 0& -0.5\end{bmatrix} +\alpha _2\begin{bmatrix}0& 0& 0\\ 0& 1& 0\\ 0& 0& -1\end{bmatrix} +\alpha _3\begin{bmatrix}0& 1& 0\\ 1& 0& 0\\ 0& 0& 0\end{bmatrix}\nonumber \\ & \quad +\alpha _4\begin{bmatrix}0& 0& 1\\ 0& 0& 0\\ 1& 0& 0\end{bmatrix} +\alpha _5\begin{bmatrix}0& 0& 0\\ 0& 0& 1\\ 0& 1& 0\end{bmatrix} \end{aligned}$$

The entire deformation process is divided into $n$ discrete steps, each with a duration of $\Delta t$. This autoregressive framework allows to model the deformation process through a series of steps, where the load at each step, $F_i$ (which can be for compression/tension, plane strain compression, or shear), governs the deformation parameter ($\phi _{F_i}$) via the macro velocity gradient (${\textbf{L}}$). The texture of the microstructure at any given step in this deformation process can be described using the ODF, as represented in Eq. (8).

8 $$\begin{aligned} \begin{aligned} A({\textbf{r}},\Delta t)&=\phi _{F_0}(A({\textbf{r}},0)) \\ A({\textbf{r}},2\Delta t)&=\phi _{F_1}(A({\textbf{r}},\Delta t)) \\ \begin{array}{c} \cdot \\ \cdot \end{array} \\ A({\textbf{r}},n\Delta t)&=\phi _{F_{n-1}}(A({\textbf{r}},(n-1)\Delta t)) \end{aligned} \end{aligned}$$

A precisely defined set of forces, ${\mathcal {F}}:=\{f_1,f_2,\ldots ,f_k\}$, is employed to select a specific force, $F_i \in {\mathcal {F}}$, for each deformation process of this physics-based simulation. The optimal final ODF set is achieved through an optimal process path, $P^*$. This path is determined by the goal of obtaining desired material properties while adhering to the ODF normalization constraint as studied previously [10].

## Software Architecture

**Fig. 2:**
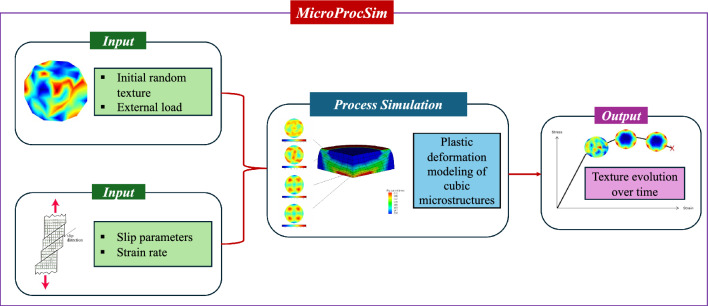
Architecture of the MicroProcSim tool that includes the input, process simulation, and software output

This physics-based deformation simulation shows how ODF changes with time under different loading conditions and varying strain rates. Therefore, the inputs for the simulator include the definition of initial texture (which may preferably be defined as randomly oriented texture or any other texture if the initial texture data is available), loading scenario, and the corresponding strain rate. It also considers the necessary slip parameters of the corresponding materials for the deformation of the cubic microstructures. The total deformation time is 0.1 sec, and the code is developed to provide the deformed ODF in every 0.01 sec time step. This allows to report the evolution of the textures during the deformations. Fig. 2 shows the architecture of MicroProcSim.

The code is designed for the cubic microstructures (FCC). According to the ODF definition, the coarse mesh for cubic microstructures involves 76 independent crystal orientations. However, this simulation provides 145 ODFs as output in each time step, including the ODFs for the dependent orientations arising due to crystallographic symmetries.

### Software Functionalities

The simulation is executed using the app.exe file provided in the project folder. The execution of this file is necessary to obtain the simulation results. The loading conditions and initial input ODFs are managed through two specific text files: ‘param.txt’ and ‘Input_ODF.txt,’ respectively. In the project folder, a MATLAB script named ‘process.m’ is written to automate the simulation for multiple runs, i.e., sequential processes. The simulation code is configured for a loading time of 0.1 s (single process). In order to run a process over 0.3 s (3 consecutive processes), the code must be executed three times sequentially, and the final ODFs from each run serve as the input for the subsequent run. The ODFs of independent crystal orientations must satisfy normalization constraints (Eq. (2)). Additionally, the ODF values cannot be negative. The MATLAB code also ensures that these conditions are met. It also saves the desired output, such as deformed ODFs and Cauchy stress tensor, at each step.

**Input File Requirements**

- The input file for loading conditions must be named ‘param.txt’ to ensure that the app.exe runs correctly. Any deviation in naming will result in the application failing to execute. Therefore, the accompanying MATLAB script is designed to consistently produce a file named param.txt to accommodate any loading condition.
- For combined loading conditions (e.g., tension and shear in the *xy*-plane), two non-zero strain rates corresponding to the respective loading conditions must be specified in the param.txt file.
- The current provided ‘input.txt’ file is only valid for randomly oriented microstructure. The user can modify this file as well to input any preferable microstructure texture.

**Output and Analysis**

- A MATLAB script, ‘process.m,’ automates multiple runs and saves the necessary output files for analysis. The script requires two .mat files, ‘newmesh.mat’ and ‘FCC_volumefraction.mat,’ to be loaded before execution. The script accommodates single or combined loading conditions over multiple runs. However, for multiple runs, the first run must be completed, and its output must be used as input for the next run with a different loading condition.
- The normalization constraint mentioned in Eq. (2) is not fully satisfied or exactly equal to 1 due to minor numerical errors. Typically, this error is less than 0.1%. However, it can propagate if multiple loading steps are performed. To mitigate this issue, the final ODF used as the initial input for the next step is normalized before applying the subsequent loading. The FCC_volumefraction.mat file helps to check the normalization constraint and update the output ODF accordingly, especially when performing multi-step loading simulations.
- This MATLAB script requires the ‘mapping.txt’ file to map the software output ODF to the ‘newmesh.mat’ ODF, as the coordinates of the ODF in the software system and MATLAB files are different. Furthermore, ‘newmesh.mat’ is utilized to plot the output ODFs in Rodrigues space. The ‘PlotFR’ function used in the MATLAB script also requires a few additional functions, which are included in this folder as well.
- Each run of the simulation generates a ‘stress–strain.out’ file containing the Cauchy Stress Tensor, which the MATLAB script also saves. Each run produces ten Cauchy Stress tensors; thus, three runs, for example, will result in thirty tensors. The Cauchy Stress tensor, originally a $3\times 3$ matrix, is converted to a column matrix in MATLAB. The file Cauchy.mat contains columns representing the Cauchy Stress Tensors, which need to be reshaped back into $3 \times 3$ matrices for further analysis.

The process can also be executed directly via ‘app.exe’ without using MATLAB for a single process. In this case, ensure that the required ‘param.txt’ and ‘Input_ODF.txt’ files are present alongside other necessary files. The folder includes five pre-configured ‘param.txt’ files for different loading conditions: tension/compression along *x*-direction, plane strain compression along *y*-direction, *xy*-shear, *xz*-shear, and *yz*-shear. To run the process for tension, for example, rename ‘param_tension.txt’ as ‘param.txt.’ and then execute ‘app.exe.’

## Illustrative Examples

This section presents example results along with the process conditions used to generate them. Three distinct cases are analyzed to explore the evolution of cubic microstructural textures: simple tension/compression, simple shear, and plane strain compression (as in a rolling process). These processes are summarized in Table 1, highlighting the three fundamental loading directions and their corresponding processing parameters ($\alpha _1$ to $\alpha _5$). A unit strain rate of $1 \, \text {s}^{-1}$ is employed across all nine cases. The framework allows for simulating various processes and strain rates by modifying the processing parameter values. This can be achieved by editing the param.txt file, either manually or using the MATLAB script process.m. The simulations were run for a total time of 0.1 seconds, divided into ten equal time steps of 0.01 seconds each, as inherently defined by the software. The initial microstructural texture was assumed to be randomly oriented, with all ODF values set equally ($A({\textbf{r}},0) \approx 2.42$), indicating an equal probability for all orientations.

**Table 1:** Loading types and corresponding parameters (in all cases, the strain rate is $1 \, \text {s}^{-1}$)

Loading type	Direction or plane	${\alpha _1}$	${\alpha _2}$	${\alpha _3}$	${\alpha _4}$	${\alpha _5}$
Tension or compression	x	1	0	0	0	0
	y	$-$ 0.5	0.75	0	0	0
	z	$-$ 0.5	$-$ 0.75	0	0	0
Shear	xy	0	0	1	0	0
	xz	0	0	0	1	0
	yz	0	0	0	0	1
Rolling or plane strain compression	zy	0	1	0	0	0
	yx	1	$-$ 0.5	0	0	0
	xz	$-$ 1	$-$ 0.5	0	0	0

**Fig. 3:**
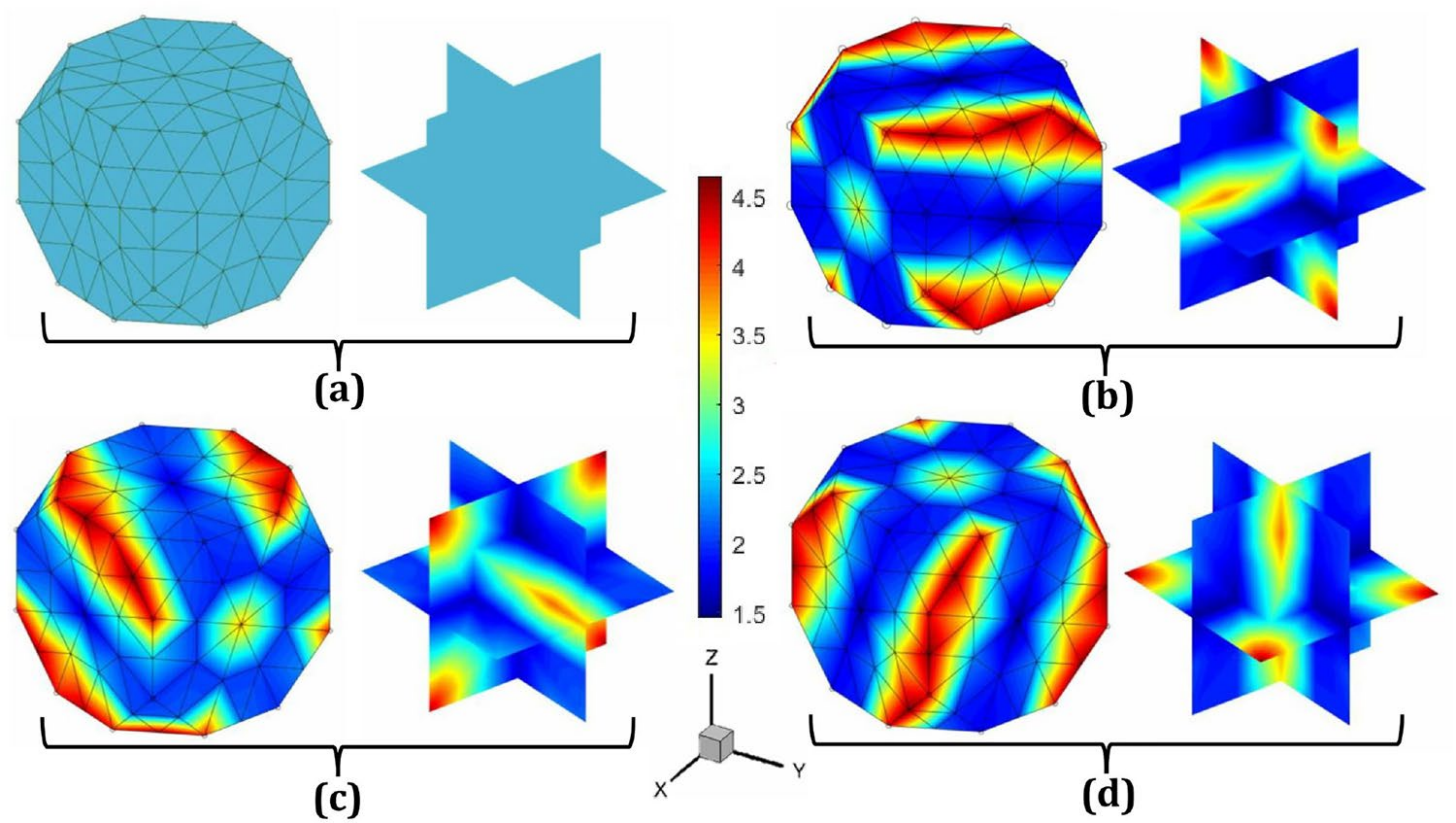
Sample microstructures on Rodrigues orientation space: **a** Initial texture with random orientation, $A({\textbf{r}},0) \approx 2.42$; final texture after applying normal strain (tension/compression) along **b** the *x*-direction, perpendicular to the *yz*-plane, **c** the *y*-direction, perpendicular to the *xz*-plane, and **d** the *z*-direction, perpendicular to the *xy*-plane. In all cases, the strain rate is $1 \, \text {s}^{-1}$, the total time is $0.1 \, \text {s}$, and the equivalent strain is $0.1 \, \text {mm}/\text {mm}$

The velocity gradient tensor, denoted as ${\textbf{L}}$, is expressed in matrix form as a $3 \times 3$ square matrix. This matrix can be tailored to enable various loading conditions and strain rates by adjusting specific processing parameters. For applying normal stress (tension/compression), the diagonal components of ${\textbf{L}}$ need to be modified appropriately. For tension or compression along the $x$-direction, perpendicular to the $yz$-plane, $L_{11}$ is set to 1, $L_{22}$ to $-$0.5, and $L_{33}$ to $-$0.5, while all other components are set to zero, preserving volume incompressibility. Similarly, for normal stress along the $y$-direction, perpendicular to the $xz$-plane, $L_{11}$ is set to $-$0.5, $L_{22}$ to 1, and $L_{33}$ to $-$0.5, with all other components zero. For normal stress along the $z$-direction, perpendicular to the $xy$-plane, $L_{11}$ is set to $-$0.5, $L_{22}$ to $-$0.5, and $L_{33}$ to 1, again ensuring volume consistency. These loading conditions can be achieved by adjusting the values of $\alpha _1$ and $\alpha _2$ as shown in the accompanying Table 1. The output microstructure textures in Rodrigues orientation space are illustrated in Fig. 3, where the strain rate is $1 \, \text {s}^{-1}$, the total time is $0.1 \, \text {s}$, and the equivalent strain is $0.1 \, \text {mm/mm}$. These simulations were performed using a single execution of the application (app.exe).

**Fig. 4:**
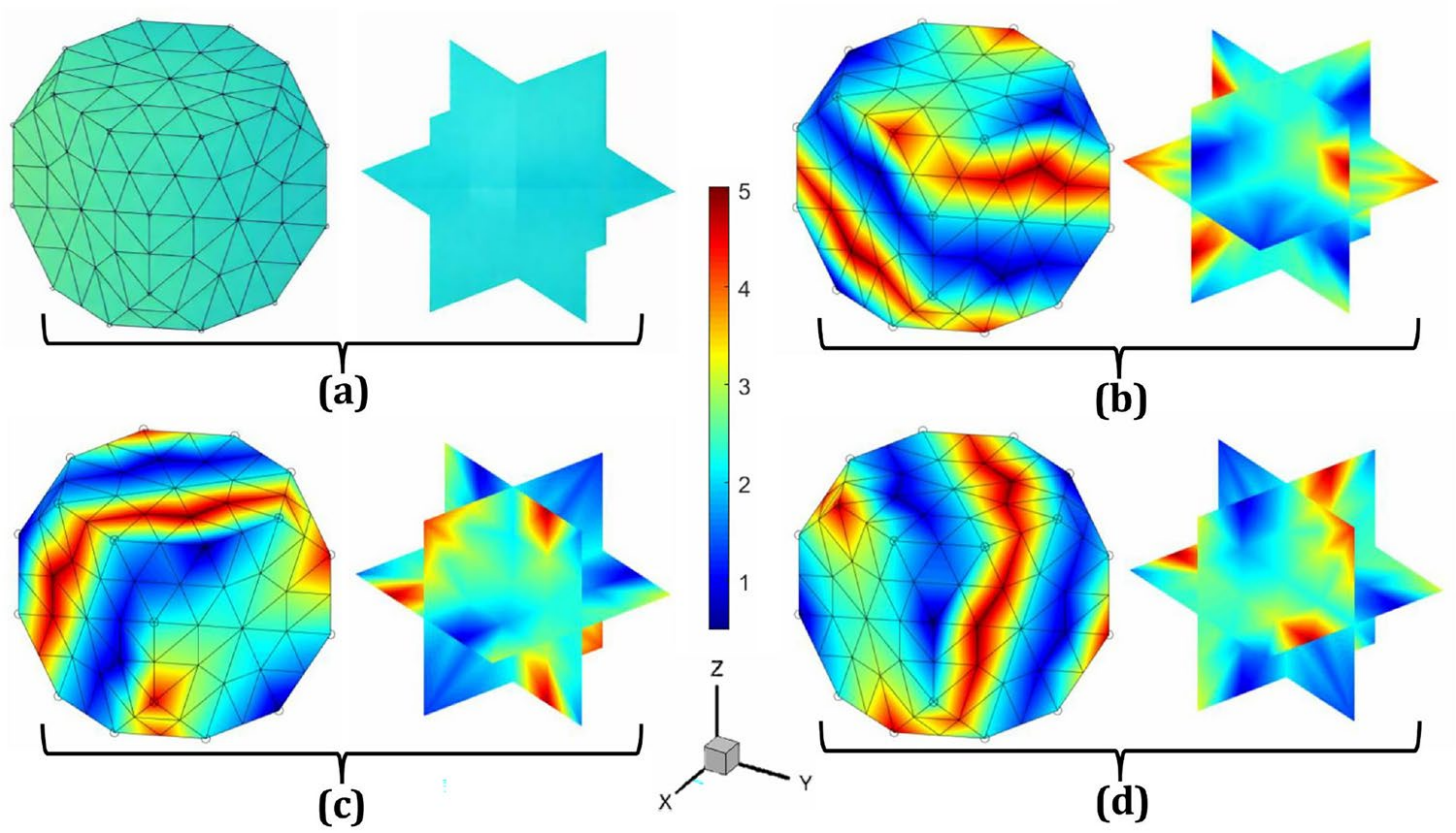
Sample microstructures on Rodrigues orientation space: **a** Initial texture with random orientation, $A({\textbf{r}},0) \approx 2.42$; final texture after applying shear strain of **b** the *xy*-shear acted on the plane perpendicular to the $x$-axis and is directed along the $y$-axis, **c** the *xz*-shear acted on the plane perpendicular to the $x$-axis and is directed along the $z$-axis, and **d** the *yz*-shear acted on the plane perpendicular to the $y$-axis and is directed along the $z$-axis. In all cases, the strain rate is $1 \, \text {s}^{-1}$, the total time is $0.1 \, \text {s}$, and the equivalent strain is $0.1 \, \text {mm}/\text {mm}$

To apply simple shear strain along the three fundamental directions ($\gamma _{xy}$, $\gamma _{xz}$, and $\gamma _{yz}$), the diagonal elements of the ${\textbf{L}}$-matrix must be set to zero, with only two off-diagonal elements set to 1. For $\gamma _{xy}$, which acts on the plane perpendicular to the $x$-axis and is directed along the $y$-axis, $L_{12} = L_{21} = 1$, while all other components are zero. This configuration ensures constant volume during the process. Similarly, for $\gamma _{xz}$, which acts on the plane perpendicular to the $x$-axis and is directed along the $z$-axis, $L_{13} = L_{31} = 1$, with the remaining components set to zero and for $\gamma _{yz}$, which acts on the plane perpendicular to the $y$-axis and is directed along the $z$-axis, $L_{23} = L_{32} = 1$, with all other components set to zero, preserving volume incompressibility. To achieve these loading conditions, only the values of $\alpha _3$, $\alpha _4$, and $\alpha _5$ need to be adjusted, as shown in the accompanying Table 1. The resulting microstructure textures in the Rodrigues orientation space are illustrated in Fig. 4, where the strain rate is $1 \, \text {s}^{-1}$, the total time is $0.1 \, \text {s}$, and the equivalent strain is $0.1 \, \text {mm/mm}$. These simulations were conducted with a single execution of the app.exe. Furthermore, due to force balance in mechanics, $\gamma _{yx}$, $\gamma _{zx}$, and $\gamma _{zy}$ are equivalent to $\gamma _{xy}$, $\gamma _{xz}$, and $\gamma _{yz}$, respectively. Thus, it is not necessary to perform separate simulations for these strain components.

**Fig. 5:**
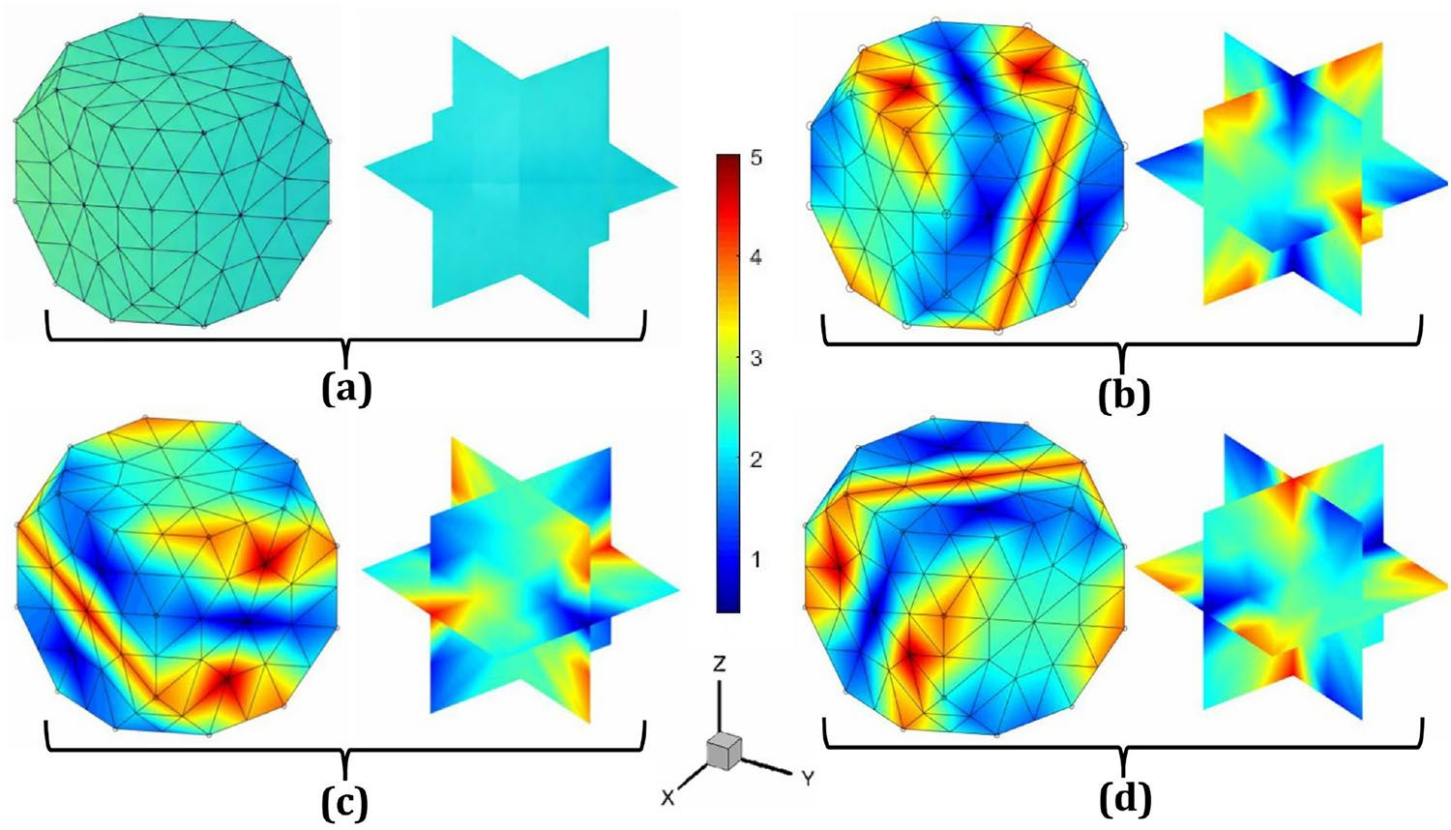
Sample microstructures in Rodrigues orientation space: **a** Initial texture with random orientation, $A({\textbf{r}},0) \approx 2.42$; final textures after the plane strain compression or rolling process along **b** the *zy*-plane, where *z* is the normal direction, *y* is the rolling direction, and *x* is the transverse direction; **c** the *yx*-plane, where *y* is the normal direction, *x* is the rolling direction, and *z* is the transverse direction; and **d** the *xz*-plane, where *x* is the normal direction, *z* is the rolling direction, and *y* is the transverse direction. In all cases, the strain rate is $1 \, \text {s}^{-1}$, the total time is $0.1 \, \text {s}$, and the equivalent strain is $0.1 \, \text {mm}/\text {mm}$

In addition to simulating microstructure evolution during the rolling process, plane strain compression can be applied in various directions equivalent to rolling by modifying the elements of the deformation gradient tensor matrix ${\textbf{L}}$. This adjustment involves ensuring that all off-diagonal elements and one diagonal element are zero, depending on the rolling direction. For instance, rolling along the $zy$-plane, where $z$ is the normal direction, $y$ is the rolling direction, and $x$ is the transverse direction, requires setting $L_{22} = 1$ and $L_{33} = -1$, which satisfies the incompressibility constraint. Similarly, for rolling along the $yx$-plane, with $y$ as the normal direction, $x$ as the rolling direction, and $z$ as the transverse direction, $L_{11} = 1$ and $L_{22} = -1$ must be set. For rolling along the $xz$-plane, where $x$ is the normal direction, $z$ is the rolling direction, and $y$ is the transverse direction, the conditions $L_{11} = -1$ and $L_{33} = 1$ ensure constant volume. These specific loading conditions can be achieved by adjusting the values of $\alpha _1$ and $\alpha _2$, as summarized in Table 1. The resulting microstructure textures on the Rodrigues orientation space are shown in Fig. 5, where the strain rate is $1 \, \text {s}^{-1}$, the total time is $0.1 \, \text {s}$, and the equivalent strain is $0.1 \, \text {mm/mm}$. Notably, these simulations were performed in a single execution of the application (app.exe).

## Extension of the Code to Different Operating Systems

**Fig. 6:**
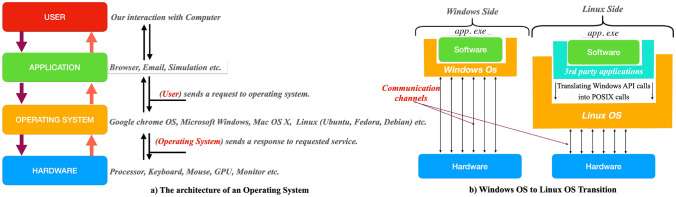
**a** The general architecture of operating systems and their message-passing mechanisms through various layers and **b** additional components required for an operating system to execute external applications that are not runnable locally

An operating system (OS) is a collection of programs that serves as an intermediary between application software and the computer hardware interface [64]. The OS is loaded by a bootloader, program, after which it facilitates the execution of hardware tasks. As illustrated in Fig. 6, communication between layers is bidirectional. By utilizing an OS, user programs have better interaction with the computer. The necessity for operating systems arises from the complexity of managing various hardware devices, including mouse, displays, and network interfaces. The OS additionally manages fundamental functions such as file systems, memory management, security, and multimedia execution. It also provides services to applications to prevent potential deadlocks and congestion [65].

In order to ensure that a program (e.g., MicroProcSim) can run on different operating systems, there are several strategies that can be used. The first option is recompiling the program with the specific libraries required for each operating system [66]. However, given the complexity of obtaining all necessary code files under present conditions, this approach may not always be feasible. An alternative solution involves creating a container [67], which provides a complete environment containing everything needed to run an application: code, runtime, system tools, system libraries, and settings. As illustrated in Fig. 6b, a program that was originally developed in a Windows operating system environment can be adapted to run in a Linux OS environment using a container. This method enables software, such as app.exe, to execute on Linux OS, even though it was originally designed for Windows.

**Fig. 7:**
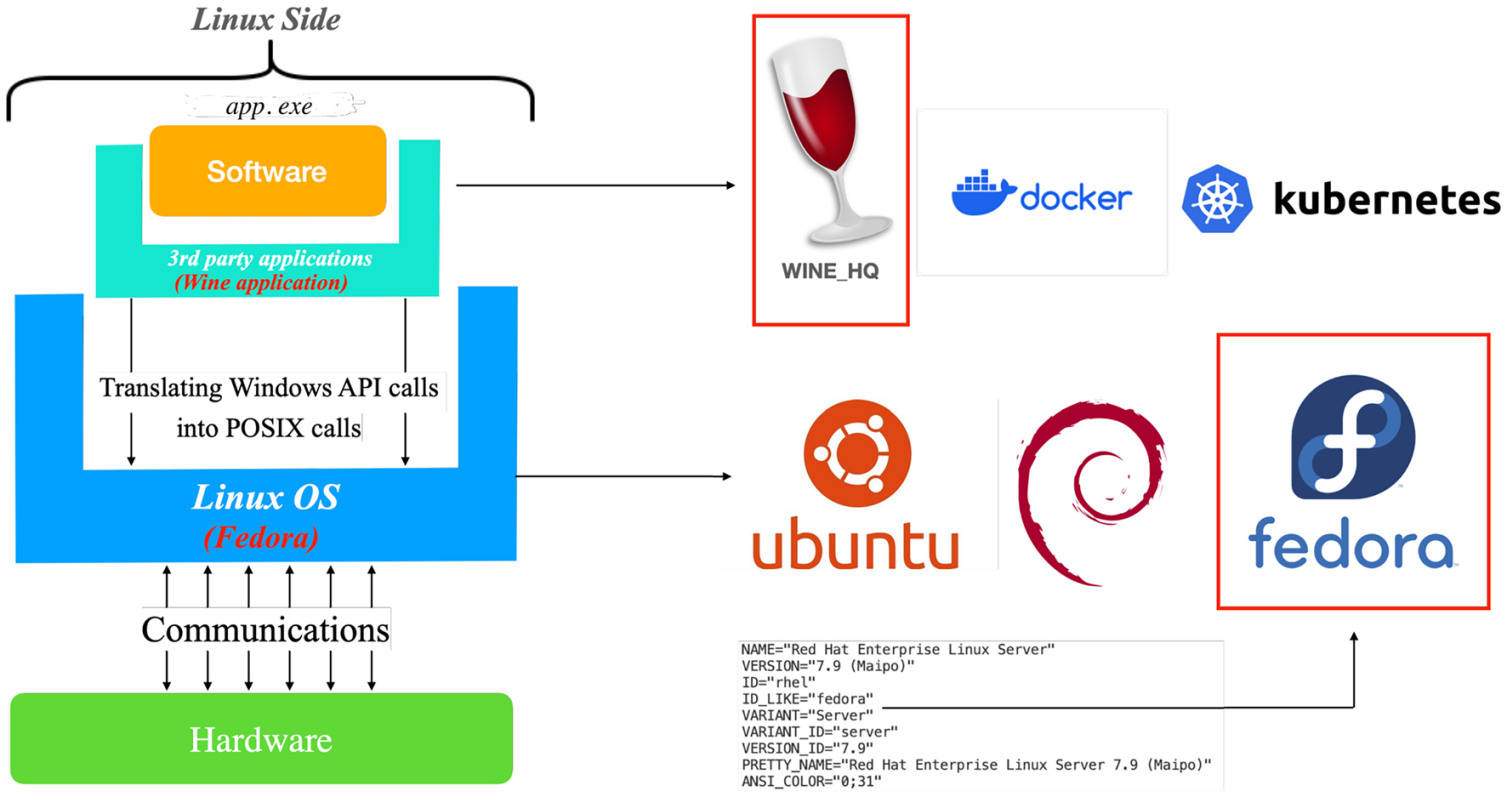
A comprehensive description of the software utilized for specific layers, along with other relevant counterparts

Different operating systems utilize various system calls, which can be considered as the operating system’s language. Examples include Windows API Calls [68] and POSIX Calls [69]. Third-party applications can facilitate the translation of these system calls from one OS to another, ensuring compatibility and functionality across diverse platforms.

In our research, we used the Windows application file of MicroProcSim called ‘app.exe,’ which was originally designed to run on a Windows operating system and make Windows API calls. To run this application on a Linux environment, we utilized a tool called Wine [70]. Wine translates Windows API calls into POSIX calls. Given the diverse internal structures of various Linux distributions, we conducted this process on a server running Fedora. Detailed information about the Fedora setup is provided in Fig. 7.

In order to use the Wine application effectively, it is important to analyze the program to be run to determine its needs, such as graphical interface, sound, networking, and serial bus activity. During the installation of Wine, these requirements should be specified as arguments in the build command. Once Wine is built, a Windows application, such as app.exe, can run as an argument of the Wine application. However, because Wine introduces an additional layer, there will inevitably be a time difference compared to running an application built natively for Linux.

## Comparative Analysis

In this section, several references will be utilized to compare the outputs from MicroProcSim with existing studies encompassing both computational and experimental investigations. Notably, most of the prior studies represent microstructures using pole figures rather than Rodrigues orientation space (ODF representation). To enable a meaningful comparison with these studies, the output ODFs have been converted into three pole figures, as required for the specific comparisons. The orientation distribution of crystals was transferred into pole figures generated for three distinct planes by utilizing the procedure of Barton et al. [71]. The pole density distribution, represented as $P({\textbf{h}}, y_i)$ describes the frequency of crystallographic orientations, where ${\textbf{h}}$ indicates the plane normal vector and $y_1, y_2, \dots , y_q$ represents specific positions on the unit sphere’s surface for the measured diffraction planes. The mathematical relationship between ODF ($A_j$) and the pole density is established through a system matrix ($M_{ij}$) by $\sum _{j=1}^k M_{ij} A_j$. This relationship accounts for *k* independent ODFs determined in the analysis. To satisfy the physical constraint that the total volume fraction must be equal to unity, the modified pole density function $\left( P_i = P_i - M_{ik}/q_k\right)$ incorporates a normalization term, with the coefficients being adjusted accordingly for the first $(k-1)$ terms, $M_{ij} = M_{ij} - M_{ik}q_j / q_k$ for $j = 1, 2, \dots , (k-1)$. However, the ODF can also be directly visualized through the pole figures using the MTEX software which is also a free and open-source toolbox widely utilized for texture analysis [72].

**Fig. 8:**
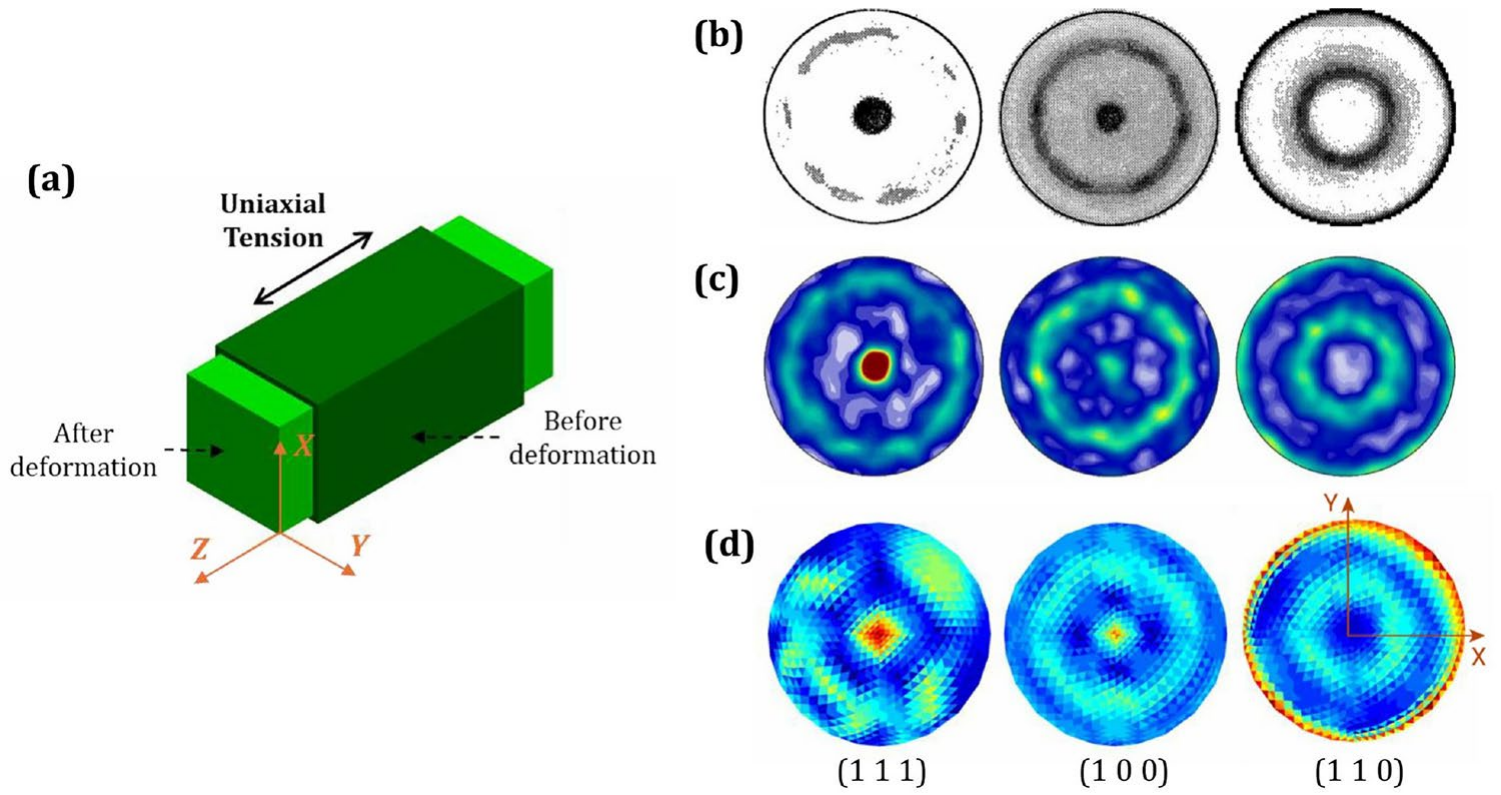
Comparison of crystallographic textures of FCC crystal in terms of (1 1 1), (1 0 0) and (1 1 0) pole figures under uniaxial tensile loading at 37% true strain: **a** Schematic representation of a sample material during the deformation process, **b** experimental pole figures obtained from oxygen-free high-conductivity (OFHC) copper specimens, as reported by Bronkhorst et al. [73], **c** simulated texture evolution for 7075-T6 aluminum alloy using PRISMS-Plasticity TM modeling software [74], and **d** simulated texture of pure copper using MicroProcSim microstructure evolution software. All microstructures exhibited initially random textures prior to deformation. ((**b**) is reprinted from Ref. [73] with permission. **c** is reprinted from Ref. [74] with permission.)

Bronkhorst et al. [73] conducted an experiment on oxygen-free high-conductivity (OFHC) copper, applying 37% true tensile strain to the randomly oriented microstructure of this FCC crystal, which exhibited isotropic properties. The final microstructure texture was documented using three distinct plane pole figures. To replicate their results, Yaghoobi et al. [74] employed the PRISMS-Plasticity TM modeling software. Instead of OFHC copper, they used the FCC 7075-T6 aluminum alloy microstructure while maintaining a similar strain level. In our study, we applied a 37% normal tensile strain to pure copper in three stages: an initial 13%, followed by 10%, and a final 10% strain ($1 - (1.13 \times 1.1 \times 1.1) \approx 37\%$ strain) along the z-direction, perpendicular to the *xy*-plane, as illustrated in Fig. 8a. To align with their methodology, we also considered an initially randomly oriented microstructure and used pole figures representing the same planes and directions, as shown in Fig. 8b–d. Analysis of the textural data reveals two primary orientational features. The presented pole figure analysis demonstrates that during tensile/compressive deformation, the polycrystalline grains undergo rotation, resulting in the alignment of either (1 1 1) or (1 0 0) crystallographic planes normal to the direction of applied stress. Furthermore, the application of simple normal strain results in an axisymmetric microstructure texture centered around the loading direction axis. The comprehensive observations derived from these pole figures indicate that the experimental and simulated microstructures were accurately captured using the presented MicroProcSim microstructure evolution software.

**Fig. 9:**
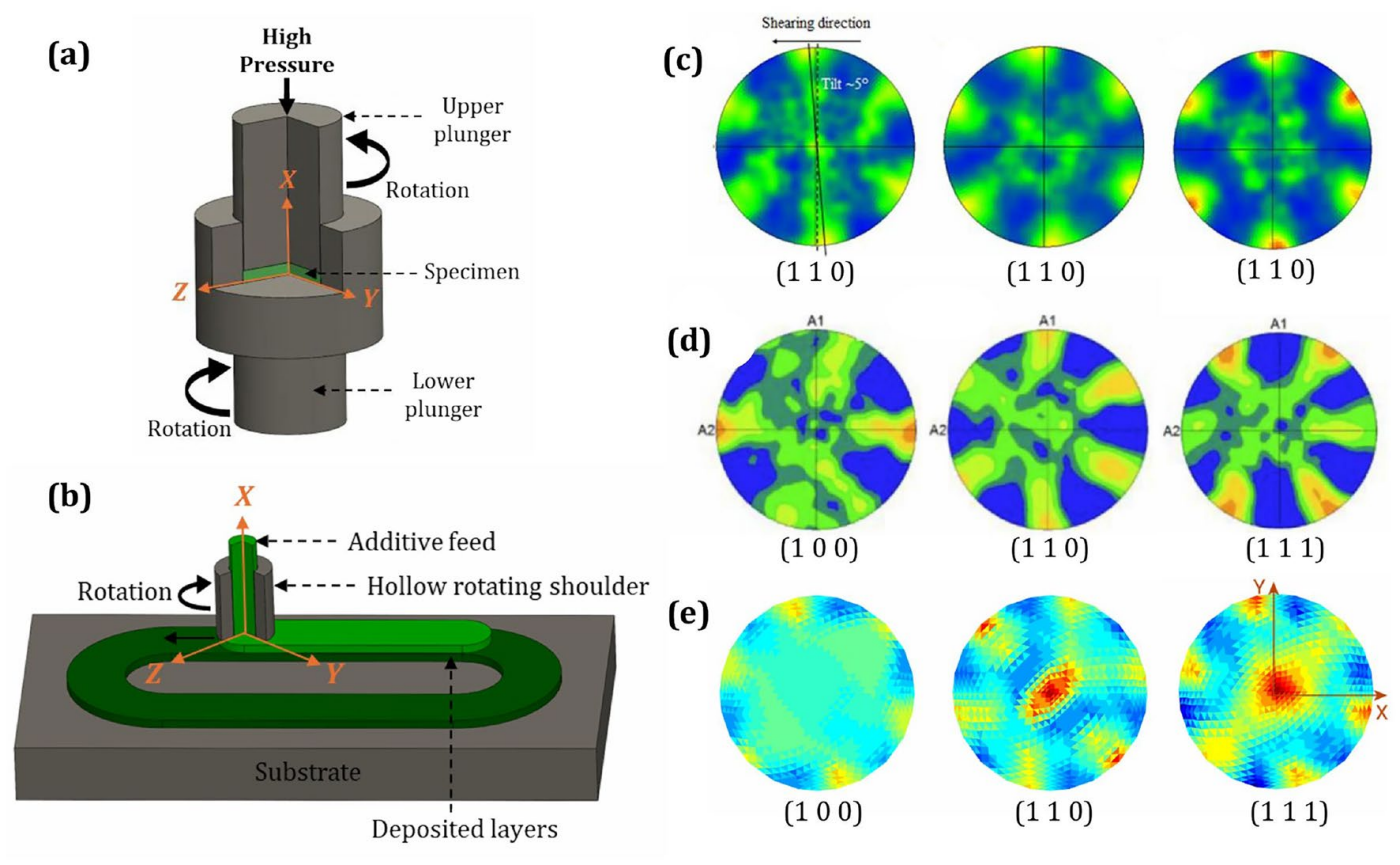
Comparison of crystallographic textures after shear processing: **a** Schematic representation of a specimen during the high-pressure torsion (HPT) deformation process; **b** schematic representation of a specimen during the additive friction stir deposition (AFSD) process; **c** experimentally observed ideal torsion texture in terms of the (1 1 0) pole figure of G91 steel alloy, (left to right) processed by HPT, followed by annealing at 600 $^\circ$C for 6 h and 24 h, as reported by Duan et al. [75]; **d** experimentally observed texture of deposited copper in terms of (1 0 0), (1 1 0), and (1 1 1) pole figures after the AFSD process, as reported by Griffiths et al. [76]; **e** simulated texture in terms of (1 0 0), (1 1 0), and (1 1 1) pole figures after simple shear on pure copper using MicroProcSim microstructure evolution software, starting from a randomly oriented initial microstructure and deformed to 0.1 mm/mm strain. ((**c**) is reprinted from Ref. [75] with permission. **d** is reprinted from Ref. [76] with permission)

To compare shear textures, the high-pressure torsion (HPT) study by Duan et al. [75] and the additive friction stir deposition (AFSD) study by Griffiths et al. [76] have been selected. Shear strain plays a critical role in both the HPT and AFSD processes. In HPT, a combination of high compressive force and torsional rotation generates intense shear deformation in the material. The strain increases radially from the center to the edges due to varying tangential displacement, as shown in Fig. 9a. This extreme deformation modifies the grain structure and texture, significantly enhancing material properties. In contrast, AFSD involves a hollow rotating tool that generates frictional heat to plasticize an additive feed material, which is then deposited onto a substrate, as illustrated in Fig. 9b. Shear strain in AFSD arises from the rotational and translational motion of the tool, causing localized deformation in the deposition zone. While HPT induces shear across the entire sample under uniform high pressure, AFSD produces localized shear to enable material flow and strong metallurgical bonding. Both processes utilize shear strain to achieve preferred grain texture refinement and improved mechanical properties. Although both shear and compressive strains exist in these processes, the shear strain magnitude is significantly higher than the compressive strain. This dominance of shear strain results in textures that resemble pure shear textures.

The HPT examination [75] analyzed both the microstructural features and textural characteristics of a Grade 91 steel containing 9% chromium. The material underwent high-pressure torsion processing followed by thermal treatment at $600^{\circ }\hbox {C}$. The pole figures of the resulting texture are represented in Fig. 9c. On the other hand, the AFSD research [76] examined how processing conditions affect microstructural development by comparing two metals with different responses to thermomechanical processing: an aluminum-magnesium-silicon alloy and pure copper. Both materials exhibit pronounced shear texture patterns. However, the study illustrates the pure copper texture in Fig. 9d for comparison with the simulated shear texture shown in Fig. 9e. To match the experimental setup, *xy*-shear was applied on the plane perpendicular to the *x*-axis and directed along the *y*-axis, as shown in Fig. 9a and b, where angular or rotational motion was applied around the *x*-axis. In the MicroProcSim simulation cases, the strain rate was set to 1 s$^{-1}$, the total simulation time to 0.1 s, and the equivalent strain to 0.1 mm/mm. The initial texture was a randomly oriented microstructure. In all the shear texture pole figures, approximately six equally spaced hotspots were observed along the circumference, as expected for shear textures if the pole figures are drawn in a manner consistent with the applied shear strain notation. The simulated texture pole figure shows reasonable agreement with the experimentally observed cubic texture after the simple shear process.

**Fig. 10:**
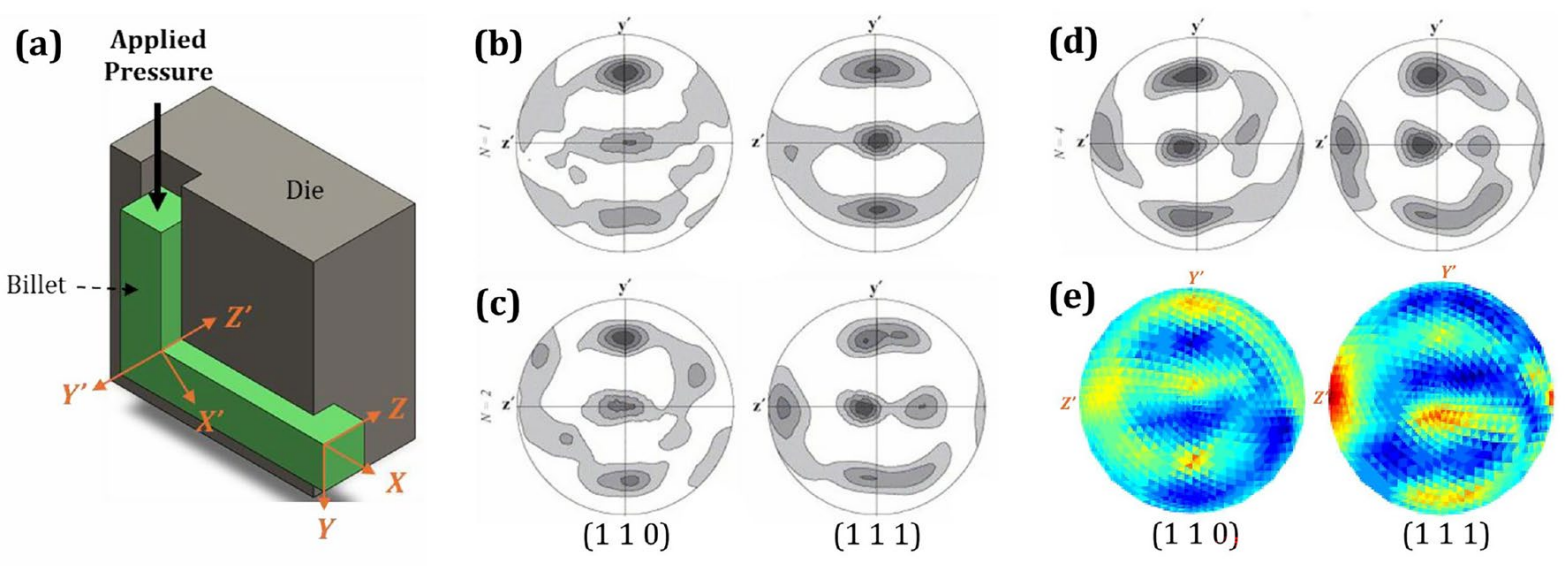
Comparison of crystallographic textures after the shear process: **a** Schematic representation of a specimen undergoing the equal channel angular extrusion (ECAE) process; **b**–**d** textures rotated by $45^{\circ }$ parallel to the shear plane of the $90^{\circ }$ ECAE die intersection, representing simple shear for IF-steel on the (1 1 0) pole figure and copper on the (1 1 1) pole figure after **b** 1 pass, **c** 2 passes, and **d** 4 passes, as reported by Gazder et al. [77]; and **e** simulated texture after simple shear on pure copper using the MicroProcSim microstructure evolution software, starting with a randomly oriented initial microstructure and deformed to 0.1 mm/mm strain. ((**b**), (**c**), and (**d**) are reprinted from Ref. [77] with permission)

Another shear deformation process is Equal Channel Angular Extrusion (ECAE), as reported by Gazder et al. [77]. This study analyzed the resulting deformed textures using an alternative axis representation which leads to differed pole figure from those in previous cases. In the ECAE process depicted in Fig. 10a, shear strain arises as the material is forced through a die with intersecting channels of equal cross section. The severe deformation occurs at the intersection of the two channels where the material must change direction sharply. As the material flows through this region under high pressure, it undergoes simple shear deformation due to the abrupt change in velocity gradient along the shear plane. This creates large plastic deformation while preserving the overall shape and cross-sectional dimensions of the billet. In this experimental investigation [77], the textures of interstitial-free (IF) steel and copper were analyzed after varying numbers of passes, as shown in Fig. 10b, c, and d. Although *xy*-shear strain occurs during the ECAE process, similar to the previous case, the reported pole figures were constructed based on the *Y* and *Z* axes instead of the *X* and *Y* axes. This shift alters the hotspot locations on the pole figures. However, texture simulation using MicroProcSim after an *xy*-shear process with a shear strain of 0.1 mm/mm, as illustrated in Fig. 10e, closely matches the experimentally observed texture when the pole figure notation is consistent with them.

**Fig. 11:**
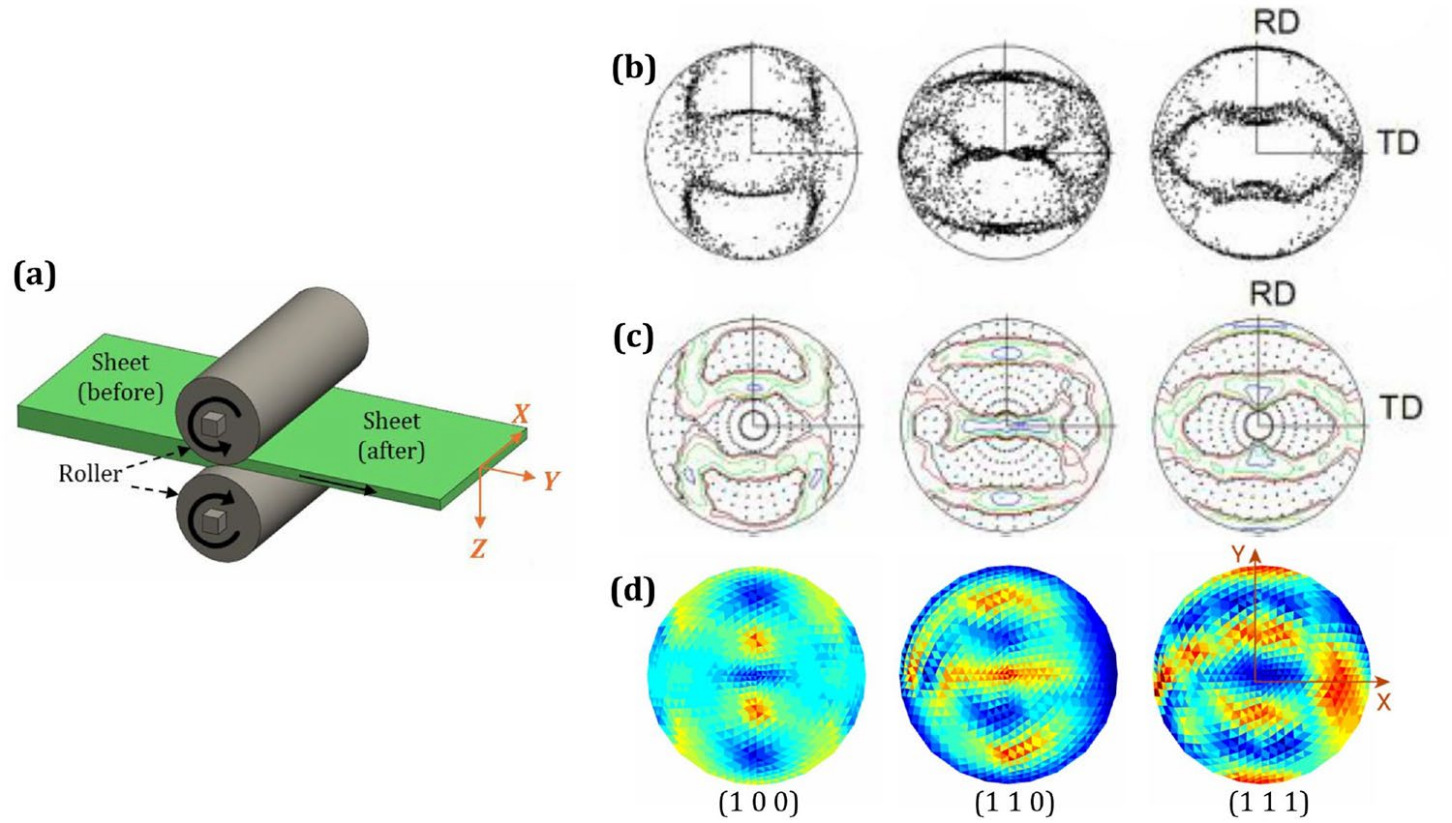
Comparison of crystallographic textures in terms of (1 0 0), (1 1 0), and (1 1 1) pole figures after plane strain compression or a rolling process with 47% reduction: **a** Schematic representation of the specimen during the rolling process; **b**–**c** equal-area pole figures of simulated rolling of an FCC aggregate, showing (**b**) dots and (**c**) intensity lines, as reported by Tomé and Lebensohn [78]; **d** simulated texture of pure copper after plane strain compression using MicroProcSim microstructure evolution software, starting from an initially random orientation. ((**b**) and (**c**) are reprinted from Ref. [78] with permission.)

Another example texture [78] after the rolling process has been compared with our MicroProcSim-simulated plane strain compression texture. In the rolling process shown in Fig. 11a, plane strain compression occurs as a metal sheet is fed through two rotating rollers. The rollers exert compressive forces in the vertical (*Z*) direction, reducing the thickness of the sheet. Since the width of the sheet (*Y* direction) remains constant due to frictional and geometric constraints, deformation primarily occurs in the thickness (*Z*) and length (*X*) directions. This restriction creates a plane strain condition where strain in the *Y* direction is negligible, resulting in a two-dimensional deformation state. The material elongates in the *X* direction while being compressed in the *Z* direction, exemplifying plane strain compression. Tomé and Lebensohn [78] simulated an FCC aggregate texture by a rolling process that involved 500 orientations, which are shown in Fig. 11b and c. In our study, plane strain compression was applied to a randomly oriented texture in four stages, where each stage had 10% strain, resulting in a total strain of approximately 47% that matches the previously reported study. The texture simulated using MicroProcSim, as shown in Fig. 11d, reasonably matches the results of the earlier work. However, the deformed texture varies among different materials due to several fundamental factors: their distinct crystal structures, available slip systems, stacking fault energies, and characteristic deformation mechanisms [79].

## Computational Costs

To demonstrate the computational cost of MicroProcSim, we have provided the execution times of the app.exe file under various loading conditions, as detailed in the illustrative examples shown in Figs. 3, 4, 5. These results are summarized in Table 2, which also includes the average utilization of memory, CPU, and GPU during the simulations. The computational analyses were conducted on a system equipped with 15.8 GB of RAM and an Intel(R) Core(TM) i7-10750 H CPU operating at a base frequency of 2.60 GHz. The system featured dual GPUs: an Intel(R) UHD Graphics processor for integrated graphics and an NVIDIA GeForce GTX 1650 Ti for high-performance tasks. For these simulations, only the Intel(R) UHD Graphics processor was utilized. Storage was provided by a PC611 NVMe SK hynix 512GB SSD, which operates on the NVMe (non-volatile memory express) protocol via a PCIe interface, enabling fast read and write operations. The machine ran on the Windows 11 Education operating system.

Table 2 lists execution times for the mentioned example simulations, which approximately range from half a minute to one minute for the given computer configurations. Eventually, these execution times mostly depend on the number of iterations required to solve the ODF, which in turn depends on the initial textures of the microstructure, type of applied load, and strain rate. The execution times for these mentioned cases were also observed when running app.exe on a Linux OS using the Wine application. In this scenario, an AMD EPYC-7702 processor with a base frequency of 2 GHz was used. The execution times vary almost linearly; for example, using Wine results in approximately 62% longer execution time for the fastest case (x-tension) and up to 123% longer for the slowest case (yz-shear). On average, the additional Wine layer causes the execution time to nearly double compared to running natively on Windows.

**Table 2:** Summary of computational costs for example simulations as illustrated in section "Illustrative Examples"

Deformation	Figs.	Execution time (s)	Average memory usage (MB)	Average CPU usage (%)	Average GPU usage (%)
x-tension	3 (b)	24.1836	13.8799	10.8636	2.2894
y-tension	3 (c)	45.0563	14.5887	21.1369	1.2874
z-tension	3 (d)	36.5485	14.2369	16.8455	1.4735
xy-shear	4 (b)	34.8552	14.0270	15.9062	2.2426
xz-shear	4 (c)	47.4494	14.0236	22.2812	2.1780
yz-shear	4 (d)	54.8004	14.0159	25.9956	1.6420
zy-rolling	5 (b)	30.1065	13.9054	13.5446	2.0764
yx-rolling	5 (c)	28.1823	14.0880	12.6850	2.4780
xz-rolling	5 (d)	38.9201	14.1903	18.0972	2.2065

When comparing MicroProcSim with recent microstructure texture evolution methods, notable differences in computational efficiency emerge. For instance, the viscoplastic self-consistent generalized material model (VPSC-GMM) [80] coupled with a Lagrangian hydrodynamics finite element code exhibits run times exceeding one minute in non-vectorized scenarios, though vectorization significantly enhances performance. These simulations addressed dynamic deformation conditions and incorporated the initial crystallographic texture of a tantalum cylinder. In contrast, PRISMS-Plasticity TM [74] demonstrates scalability advantages: weak-scaling tests for a polycrystalline copper sample with 400–102400 grains on 256 processors achieved a wall time of $\sim$400 s, while strong-scaling analyses of a 400 grain sample under 100% compressive strain showed wall times of 8 s on 64 processors and 100–200 s when using fewer processors (e.g., 4 or 16). Notably, MicroProcSim demonstrates lower simulation costs compared to both existing crystal plasticity texture evolution software and other texture evolution modeling approaches.

## Conclusion

The development of MicroProcSim marks a significant advancement in the simulation of metallic microstructures under deformation processes. MicroProcSim effectively predicts the evolution of microstructural textures in terms of ODFs under various loads and strain rates. This tool, originally designed for Windows and now extended to Linux, offers a robust solution for replicating the deformation behavior of cubic microstructures. It saves significant time and resources, which are otherwise typically spent on experimental observations. A MATLAB code is also included in the software package to automate the process for consecutive processing and save the desired output. In this study, sample results are reported for different loading conditions. In contrast to conventional crystal plasticity finite element software, MicroProcSim stands out by swiftly generating deformed textures without accounting for grain morphology, focusing solely on grain texture. Additionally, comparisons with experimental and computational studies on texture evolution confirm that the software effectively mimics real-world material processing conditions with just a simple adjustment to a single input matrix. This simulation tool will provide engineers and researchers with a reliable method for understanding and predicting the large deformation behavior of materials, with the potential to contribute to more informed decision-making and the development of more resilient materials. The future work on MicroProcSim will include the utilization of GPU resources to further improve its computational efficiency, development of a user-friendly graphical interface, as well as the extension of the microstructure formulation to different crystallographic systems (Table 3).

**Table 3:** Code metadata

Code metadata	Description
Current code version	*v1.0*
Permanent link to code/repository used for this code version	https://github.com/NU-CUCIS/MicroProcSim
Legal code license	GNU General Public License (GPL)
Software code languages, tools, and services used	C++, Matlab
Compilation requirements, operating environments, and dependencies	Windows Linux (with Wine application)
If available, link to developer documentation/manual	https://github.com/NU-CUCIS/MicroProcSim/blob/main/README.md
Support email for questions	pacar@vt.edu

## Data Availability

The GitHub access link of MicroProcSim along with other code metadata can be found in the table above.
